# Association Between Urinary Protein-to-Creatinine Ratio and Chronic Kidney Disease Progression: A Secondary Analysis of a Prospective Cohort Study

**DOI:** 10.3389/fmed.2022.854300

**Published:** 2022-03-31

**Authors:** Xun Qin, Haofei Hu, Ji Cen, Xiaoyu Wang, Qijun Wan, Zhe Wei

**Affiliations:** ^1^Department of Nephrology, Hechi People's Hospital, Hechi, China; ^2^Department of Nephrology, Shenzhen Second People's Hospital, Shenzhen, China; ^3^Department of Nephrology, The First Affiliated Hospital of Shenzhen University, Shenzhen, China; ^4^Shenzhen University Health Science Center, Shenzhen, China

**Keywords:** urinary protein-to-creatinine ratio, non-linearity, chronic kidney disease progression, Cox proportional hazards regression, linear mixed-effects regression model

## Abstract

**Objective:**

Studies on the association between urinary protein-to-creatinine ratio (UPCR) and chronic kidney disease (CKD) progression are limited. This study aimed to investigate the relationship between UPCR and CKD progression in a Japanese population.

**Methods:**

The present research was a secondary analysis of a prospective cohort study. Eight hundred and ninety-six subjects from the research of CKD-ROUTE in Japan were included. All the patients were new visitors or first referred to the participating centers of nephrology between October 2010 and December 2011. The target-independent variable was UPCR measured at baseline. The dependent variable was CKD progression and the estimated glomerular filtration rate (eGFR) changes during follow-up. We used Cox proportional hazards regression to investigate the association between UPCR and CKD progression risk. To address UPCR and CKD progression's non-linearity, a multivariate Cox proportional hazards regression analysis with cubic spline functions model and smooth curve fitting (penalized spline method) were conducted. We further used a generalized linear mixed model to explore the relationship between UPCR and the changes of eGFR.

**Result:**

The mean age of the included patients was 67.2 ± 13.4 years old. Two hundred and thirty-four people occurred CKD progression during follow-up. The present study showed that UPCR was independently associated with CKD progression in the multivariate analysis [HR = 1.164, 95% CI (1.116, 1.215)]. The non-linear relationship between UPCR and CKD progression was explored in a dose-dependent manner, with an obvious inflection point of 1.699. Furthermore, our findings indicated that the tendency of the effect sizes on both the left and right sides of the inflection point was not consistent [left HR: 4.377, 95% CI (2.956, 6.483); right HR: 1.100, 95% CI (1.049–1.153)]. Using the linear mixed-effects regression model, we found that UPCR was an independent predictor of the longitudinal changes in eGFR (*p* < 0.001 for the interaction term with time).

**Conclusion:**

This study demonstrates a nonlinear positive relationship between UPCR and CKD progression in the Japanese population. UPCR is also an independent predictor of the longitudinal changes in eGFR.

## Background

Chronic kidney disease (CKD), resulting in end-stage renal disease (ESRD), has become a significant health problem worldwide. In recent years, billions of dollars have been charged to the National Health Insurance system, and the cost has continued to rise (1). CKD affects about 14% of the United States population, and the prevalence of ESRD is around 2,043 per 1 million people, which is ranked third in the world (2). There are ~13.3 million people, accounting for 13% of the Japanese adult population, are estimated to have CKD (3). In addition, patients with CKD also have poorer cardiovascular outcomes and higher mortality (4). Therefore, studying the risk factors that may lead to the damage and deterioration of renal function has become the top priority of preventing and treating kidney diseases.

Diabetes mellitus (DM), age, gender, dyslipidemia, anemia, high-protein diet, smoking, obesity, hyperuricemia, proteinuria, family history for CKD and hypertension are traditional risk factors for the development of CKD (5, 6).

Proteinuria not only indicates the severity of CKD but is also strongly related to CKD progression (7). It is therefore essential to evaluate accurately each patient's proteinuria levels. In clinical practice, there are three indicators used to assess proteinuria: 24-h urine protein excretion (UPE), protein-to-creatinine ratio (UPCR), and urinary albumin-creatinine ratio (ACR). Although 24-h UPE is the most commonly used measure of proteinuria in randomized controlled clinical trials, it has several limitations and is unreliable if not validated by measuring 24-h urinary creatinine concomitantly. It is inconvenient and often inaccurate due to the 24-h urine collection required, and the quality of the urine is easily affected by the environment (8). Some studies support recommendations of using spot UPCR in screening and monitoring proteinuria in CKD patients (8), such as nephritis (9), diabetic nephropathy (10), and IgA nephropathy (11). Currently, a few studies have explored the relationship between UPCR and CKD progression (11–13). Nevertheless, most of these studies only used the logistic regression model or Cox proportional hazards regression to explore the relationship between UPCR and CKD progression. Therefore, evidence on the quantitative relationship between UPCR and CKD progression is still limited. Besides, few studies have investigated the relationship between baseline UPCR and the changes in estimated glomerular filtration rate (eGFR) and the role of other variables in modifying the relationship between UPCR and CKD progression. Moreover, fewer studies evaluated the possible non-linear relationship between UPCR and CKD progression.

Therefore, this study would use a multivariate Cox proportional hazards regression analysis, a multivariate Cox proportional hazards regression analysis with cubic spline functions model and smooth curve fitting (penalized spline method), and a generalized linear mixed model to investigate whether the baseline UPCR was independently related to renal function progression and the changes of eGFR in patients with CKD. A comprehensive understanding of the relationship between UCPR and the risk of CKD progression and changes in eGFR can provide a more scientific reference for clinically delaying renal function progression in CKD patients by controlling proteinuria.

## Methods

### Data Source and Participants

Data could be downloaded from the “DATADRYAD” database (www.datadryad.org), a website that allowed users to download raw data freely. All occurrence data for specimens included in this study were available as part of a Dryad (http://datadryad.org/) data package (doi: 10.5061/dryad.kq23s) (14). The variables used in the study were as follows: gender, age, systolic blood pressure (SBP), body mass index (BMI), serum creatinine (Scr), UPCR, urinary occult blood, eGFR, hemoglobin (Hb), serum albumin (ALB), causes of CKD, history of cardiovascular disease (CVD), diabetes, hypertension and anti-hypertensive therapy including calcium channel blocker, angiotensin receptor blockers (ARB), angiotensin-converting enzyme inhibitors (ACEI), and diuretics, years of follow up and CKD progression at follow up (14). According to the Dryad Terms of Service, researchers might apply these data in secondary analysis without infringing on the authors' rights. As written informed consent and research ethics approved were obtained in the previous research, no longer needed for this secondary study (14).

Data were obtained from the research on chronic kidney disease outcomes in treatment and epidemiology (CKD-ROUTE), a prospective, observational cohort study of a representative Japanese population with stage G2-G5 CKD. Stage of CKD was defined based on Kidney disease: improving global outcomes (KDIGO) classification (15). Details of the design in the study have been reported previously (14, 16, 17). More than 1,000 participants participated in Tokyo Medical and Dental University Hospital, and its 15 affiliated hospitals were enrolled (14).

**Figure 1:**
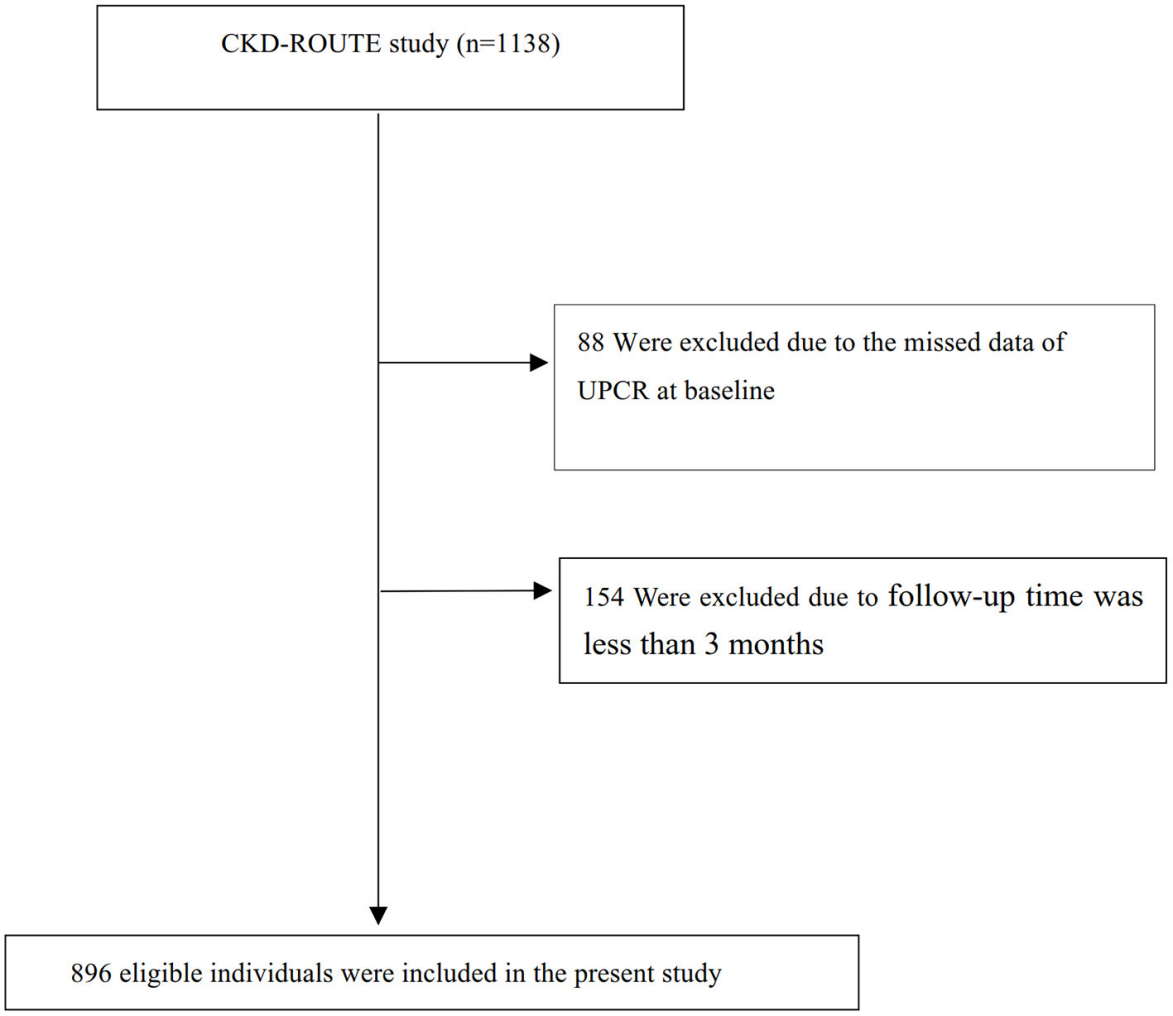
Flowchart of study participants.

New patients who were older than 20 years of age and who visited or were referred for the treatment of CKD stage 2–5 between October 2010 and December 2011, but were not on dialysis therapy, were recruited in this study (14). Patients with malignancy, transplant recipients, and/or active gastrointestinal bleeding; those who did not provide informed consent were excluded (14). Finally, 1,138 patients were assessed for eligibility in the original study (14). We excluded patients with missing values of UPCR (*n* = 88), and follow-up time was <3 months (*n* = 154). The final analysis included 896 subjects (629 male and 267 female) in the present study (see flowchart for details in Figure 1).

### Study Design and Measurement of Variables

This was a prospective cohort study, and the study's design had been documented elsewhere (14, 17). All patients' medical history and current medications were recorded at enrollment. BMI was calculated from the body height and weight, obtained by anthropometric measurements. A standard sphygmomanometer was used to measure BP. Urine and blood samples were collected to measure creatinine, hemoglobin, albumin, urinary protein, urinary occult blood, and urinary creatinine (14). The eGFR was calculated by the following diet modification in renal disease equation modified for Japanese subjects (18): eGFR = 194 × serum creatinine ^−1.094^ × age ^−0.287^ (if female, × 0.739). Proteinuria was identified by urine dipstick test and the UPCR. Anemia was defined as hemoglobin level <10 g/dL because treatment for anemia was provided with a target Hb level of 10–12 g/dL. Low BMI (<23.5 kg/m^2^) and low serum albumin level (<4 g/dL) were defined as cutoff values (19). All patients received standard treatment protocols according to the Japanese CKD guidelines (20). All patients were visited every 6 months for assessment of their clinical status. UPCR at baseline was the main independent variable. The dependent variable was CKD progression and eGFR changes during the follow-up period.

### Definition of Diabetes, Hypertension, Cardiovascular Disease, and Etiology of Kidney Disease

Hypertension was defined as SBP at least 140 mmHg or DBP at least 90 mmHg or clinician-diagnosed hypertension, or currently on anti-hypertensive medication (14). Diabetes mellitus was defined as HbA1c ≥ 6.5% or antidiabetic therapy history (14). The etiology of CKD in each patient was determined by the physician who treated the patient at the time of enrollment, according to the patient's clinical characteristics, past medical history, and histological findings of renal biopsy specimens (14).

CVD was defined as having a history of coronary heart disease (including myocardial infarction, angina pectoris, coronary revascularization), congestive heart failure, peripheral arterial disease, or stroke (transient ischemic attack, cerebral infarction, subarachnoid hemorrhage, or cerebral hemorrhage) (14).

### Study Endpoint

The primary outcome was CKD progression, defined as either initiation of dialysis during or 50% decline in eGFR from baseline (14). And the secondary endpoint was the changes of eGFR.

### Statistical Analysis

First, we dealt with the missing values of covariates. The number of participants with missing SBP, BMI, and ALB data was 13, 89 and 3, respectively. Since only some continuous variables had missing data and were not much, it was unlikely to make a big impact. They all satisfied the normal distribution, so we used means to supplement the missing data (21).

Next, UPCR tertiles stratified baseline characteristics of all patients. Continuous variables with normal and skewed distribution were expressed as means with standard deviations or medians with interquartile ranges. Categorical variables were expressed as numbers and percentages. To compare differences among different UPCR groups (tertiles based on UPCR data), one-way analysis of variance (parametric distribution) or Kruskal–Wallis (non-parametric distribution) test was applied. Chi-square was used to compare categorical variables. Kaplan-Meier survival analysis with the log-rank test was used to compare CKD progression-free survival of different UPCR groups.

The process of data analysis was based on two criteria: (1) what was the real relationship between UPCR and CKD progression (linear or non-linear); (2) adjusted the confounding variables or after the stratified analysis, what was the true relationship between UPCR and CKD progression? Therefore, all of the results presented in this study were based on a three-step data analysis approach. Step 1: Univariate and multivariate Cox proportional hazard regression models were employed. According to the recommendation of the STROBE statement (22), we constructed three models: model 1, no covariates were adjusted; model 2, only adjusted for sociodemographic data (age, BMI, gender, SBP, hypertension, diabetes, history of CVD, and etiology of CKD); model 3, model 2+other covariates (HB, eGFR, ALB, urinary occult blood, use of calcium channel blocker, use of RAAS inhibitor, and use of diuretics). When added to the model, those covariates that changed the coefficient by more than 10% were considered confounders and adjusted for the multivariate analysis (23). Step 2: To address the non-linearity of UPCR and CKD progression, a Cox proportional hazards regression with cubic spline functions and smooth curve fitting (the cubic spline smoothing) were conducted. We calculated the inflection point using a recursive algorithm to detect a non-linearity. Then a two-piecewise Cox proportional hazard model was performed to calculate the threshold effect of the UPCR on CKD progression in terms of the smoothing plot. In the end, which model was more suitable for fitting the association between UPCR and CKD progression was mainly determined by the log-likelihood ratio test.

A sensitivity analysis was conducted to ensure the robustness of the data analysis. UPCR was converted into a categorical variable, and the *P*-value was calculated for the trend. The test's purpose was to verify the results of treating UPCR as a continuous variable and determine the possibility of non-linearity. Achieving complete remission predicts an excellent long-term renal prognosis (24). Therefore, when exploring the association between UPCR and CKD progression in other sensitivity analyses, we excluded participants with UPCR < 0.3.

Furthermore, eGFR levels were followed every 6 months until 36 months. The longitudinal changes in eGFR were analyzed with linear mixed-effects regression models (25), which easily accommodate unbalanced, unequally spaced observations (26). The dependent variable (i.e., eGFR) was assessed on the baseline visit and during all follow-up visits in these models. In contrast, the independent variable (i.e., UPCR) was only measured on the baseline visit. The following variables, measured or calculated on the baseline visit, were entered into all of the models as fixed effects: age, BMI, gender, SBP, hypertension, diabetes, history of CVD, etiology of CKD, HB, eGFR, ALB, urinary occult blood, use of calcium channel blocker, use of RAAS inhibitor, and use of diuretics. In mixed-effects regression models, the interaction term between a fixed effect variable and time assessed whether the variable was a predictor of longitudinal changes in the eGFR variable. Therefore, the interaction terms between time and UPCR were evaluated. All models also included intercept as random term. Random effects allowed each participant's beginning value to vary from the population average (intercept). Patient level (each patient has one intercept) was the level of random intercept. Since patients were assessed for eGFR every 6 months for a total of 6 times, time to repeated measures was treated as a categorical variable (0, 6, 12, 18, 24, 30, 36 months). No structure was imposed on the covariance matrix of these random effects, and the errors were assumed to be independent with constant variance.

All analyses were performed using R (http://www.R-project.org) and EmpowerStats software (www.empowerstats.com, X&Y solutions, Inc., Boston, MA, USA). *P*-values <0.05 were considered statistically significant.

## Results

Thus, a total of 895 participants (70.2% men and 29.8% women) were eventually included in this analysis. The mean age was 67.2 years old (SD = 13.4). The mean follow-up time was 26.53 ± 11.83 months, and 234 people developed CKD progression during follow-up. The baseline mean UPCR and eGFR were 2.09 ± 3.22 and 33.19 ± 17.97 ml/min per 1.73 m^2^, respectively.

### Baseline Characteristics of the Study Participants

Baseline characteristics of the study population were presented by tertiles of UPCR (Table 1). We divided participants into subgroup using UPCR tertiles (<0.224, 0.22–1.648, ≥1.648). In the highest UPCR group, we found that patients generally had higher Scr, SBP, BMI levels, higher rates of urinary occult blood, diabetes, hypertension, history of CVD, calcium channel blocker use, RAAS inhibitor use, and use of diuretics. Besides, patients in the highest UPCR group had a higher proportion of diabetic nephropathy as the primary disease. In contrast, patients generally had lower age, HB, ALB, and eGFR levels in the highest UPCR group.

**Table 1:** Baseline characteristics of all the patients at enrollment (*n* = 896).

**UPCR**	**T1 (<0.224)**	**T2 (0.224–1.648)**	**T3 (≥1.648)**	***P*-value**
Participants	299	298	299	
Age (years)	68.44 ± 13.10	67.67 ± 13.64	65.46 ± 13.35	0.019
HB (g/dL)	12.91 ± 1.97	12.05 ± 2.28	11.31 ± 2.06	<0.001
Scr (mg/dL)	1.29 (1.08–1.72)	1.70 (1.20–2.50)	2.40 (1.68–3.45)	<0.001
eGFR (ml/min per 1.73 m^2^)	41.56 ± 15.50	33.31 ± 18.23	24.69 ± 15.98	<0.001
UPCR (g/gCr)	0.07 (0.03–0.13)	0.70 (0.40–1.04)	4.22 (2.63–6.90)	<0.001
SBP(mmHg)	132.63 ± 19.30	138.19 ± 21.15	148.83 ± 22.62	<0.001
BMI(kg/m^2^)	23.59 ± 3.39	23.70 ± 3.68	24.32 ± 4.26	0.039
ALB(g/dL)	4.20 ± 0.43	3.99 ± 0.47	3.44 ± 0.63	<0.001
Gender				0.439
Male	216 (72.24%)	211 (70.81%)	202 (67.56%)	
Female	83 (27.76%)	87 (29.19%)	97 (32.44%)	
Etiology of CKD				<0.001
Diabetic nephropathy, *n* (%)	24 (8.03%)	48 (16.11%)	160 (53.51%)	
Nephrosclerosis, *n* (%)	177 (59.20%)	121 (40.60%)	59 (19.73%)	
Glomerulonephritis, *n* (%)	23 (7.69%)	80 (26.85%)	60 (20.07%)	
Other, *n* (%)	75 (25.08%)	49 (16.44%)	20 (6.69%)	
Urinary occult blood, *n* (%)	50 (16.72%)	98 (33.11%)	142 (47.49%)	<0.001
Hypertension, *n* (%)	242 (80.94%)	272 (91.28%)	292 (97.66%)	<0.001
History of CVD, *n* (%)	65 (21.74%)	75 (25.17%)	101 (33.78%)	0.003
Diabetes, *n* (%)	78 (26.09%)	89 (29.87%)	178 (59.53%)	<0.001
Use of RAAS inhibitor, *n* (%)	173 (57.86%)	187 (62.75%)	226 (75.59%)	<0.001
Use of calcium channel blocker, *n* (%)	100 (33.44%)	150 (50.34%)	181 (60.54%)	<0.001
Use of diuretics, *n* (%)	69 (23.08%)	84 (28.19%)	138 (46.15%)	<0.001

*Continuous variables are presented as mean ± standard deviation and median with interquartile ranges. Categorical data are presented as numbers and percentages*.

*BMI, body mass index; SBP, Systolic blood pressure; Scr, Serum creatinine; ALB, Serum albumin; HB, Hemoglobin; CKD, chronic kidney disease; CVD, cardiovascular disease; eGFR, estimated glomerular filtration rate; UPCR, urinary protein/creatinine ratio; g/gCr, gram per gram creatinine; RAAS, renin-angiotensin aldosterone system*.

**Figure 2:**
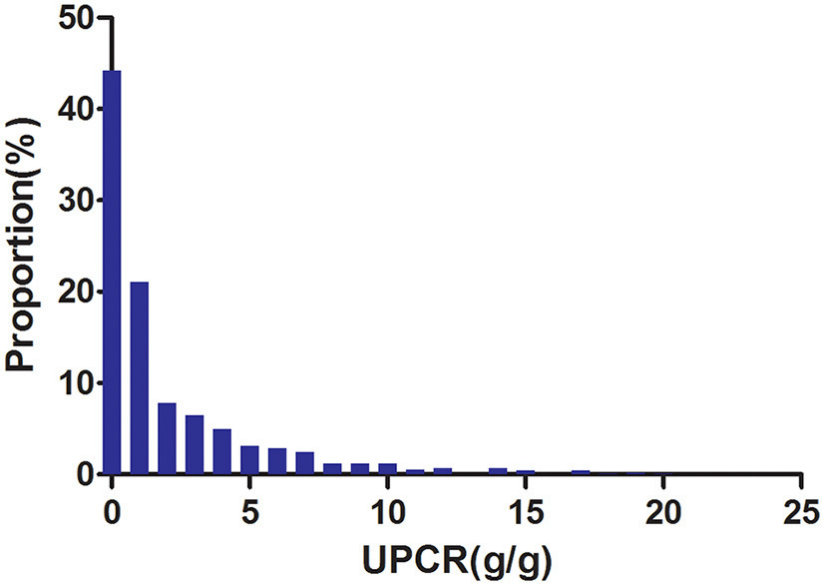
Distribution of UPCR. It presented a skewed distribution while being in the range from 0.006 to 20.183.

**Figure 3:**
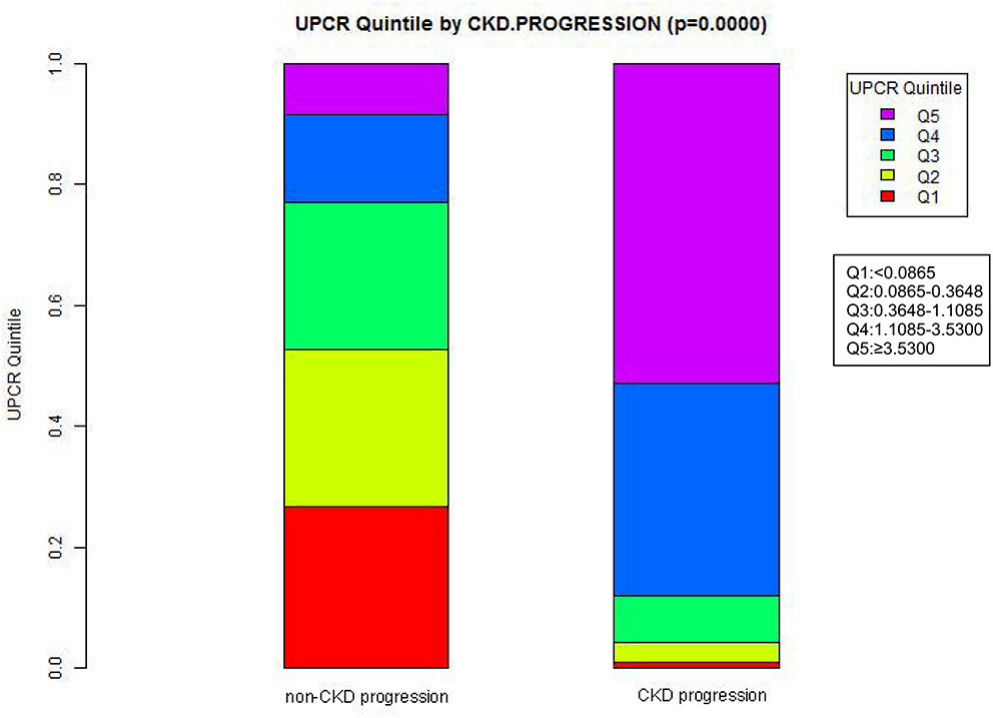
Data visualization of UPCR of all participants from CKD progression and non-CKD progression groups. The results indicated that the distribution level of UPCR in the CKD progression group was higher, while the level of UPCR in the CKD progression-free group was relatively low.

Figure 2 showed the distribution of UPCR levels. It presented a skewed distribution while being in the range from 0.006 to 20.183. This result also indicated that most of the FLI were <6.329. Participants were divided into two groups according to whether they developed CKD progression during the follow-up. The UPCR values in the two groups were shown in Figure 3. The results indicated that the distribution level of UPCR in the CKD-progression group was higher, while the UPCR level in the CKD-progression-free group was relatively lower.

### The Incident Rate of CKD Progression

Table 2 revealed that 234 patients developed CKD progression in total. The total incident rate of all participants was 11.814 per 100 person-years. Specifically, the incident rates of the three UPCR groups were 0.957, 5.776, and 35.424 per 100 person-years, respectively.

**Table 2:** Incident rate of incident CKD pregression.

**UPCR**	**Participants (*n*)**	**CKD progression** **event (*n*)**	**Incident rate** **(Per 100 person-year)**
Total	896	234	11.814
T1	299	7	0.957
T2	298	42	5.776
T3	299	185	35.423

### Univariate Analysis

The results of the univariate Cox regression analysis were shown in Table 3. The results showed that UPCR, urinary occult blood, SBP, diabetes, hypertension, use of calcium channel blocker, use of RAAS inhibitor, and use of diuretics were positively associated with the risk of CKD progression. In contrast, HB, eGFR and ALB were negatively related to the risk of CKD progression. We also found that patients with primary onset diabetic nephropathy had a high risk of CKD progression.

**Table 3:** The results of univariate analysis.

	**Statistics**	**Effect size HR (95% CI)**	***P*-value**
Age	67.19 ± 13.41	0.99 (0.98, 1.00)	0.1350
**Gender**
Male	629 (70.20%)	Ref.	
Female	267 (29.80%)	1.05 (0.79, 1.38)	0.7405
**Etiology of CKD**
Diabetic nephropathy	232 (25.89%)	Ref.	
Nephrosclerosis	357 (39.84%)	0.17 (0.13, 0.24)	<0.0001
Glomerulonephritis	163 (18.19%)	0.27 (0.19, 0.39)	<0.0001
Other	144 (16.07%)	0.16 (0.10, 0.26)	<0.0001
HB	12.09 ± 2.20	0.70 (0.66, 0.74)	<0.0001
eGFR	33.18 ± 17.97	0.92 (0.91, 0.93)	<0.0001
**Urinary occult blood**
No	604 (67.56%)	Ref.	
Yes	290 (32.44%)	1.72 (1.32, 2.22)	<0.0001
UPCR	2.09 ± 3.22	1.21 (1.18, 1.24)	<0.0001
**Hypertension**
No	90 (10.04%)	Ref.	
Yes	806 (89.96%)	5.44 (2.24, 13.19)	0.0002
**History of CVD**
No	655 (73.10%)	Ref.	
Yes	241 (26.90%)	1.25 (0.94, 1.66)	0.1211
**Diabetes**
No	551 (61.50%)	Ref.	
Yes	345 (38.50%)	2.87 (2.21, 3.72)	<0.0001
**Use of RAAS inhibitor**
No	310 (34.60%)	Ref.	
Yes	586 (65.40%)	1.75 (1.30, 2.37)	0.0003
**Use of calcium channel blocker**
No	465 (51.90%)	Ref.	
Yes	431 (48.10%)	1.77 (1.36, 2.30)	<0.0001
**Use of diuretics**
No	605 (67.52%)	Ref.	
Yes	291 (32.48%)	2.29 (1.77, 2.96)	<0.0001
SBP	139.88 ± 22.09	1.02 (1.01, 1.02)	<0.0001
BMI	23.87 ± 3.80	1.03 (0.99, 1.07)	0.0973
ALB	3.88 ± 0.61	0.35 (0.30, 0.41)	<0.0001

**Figure 4:**
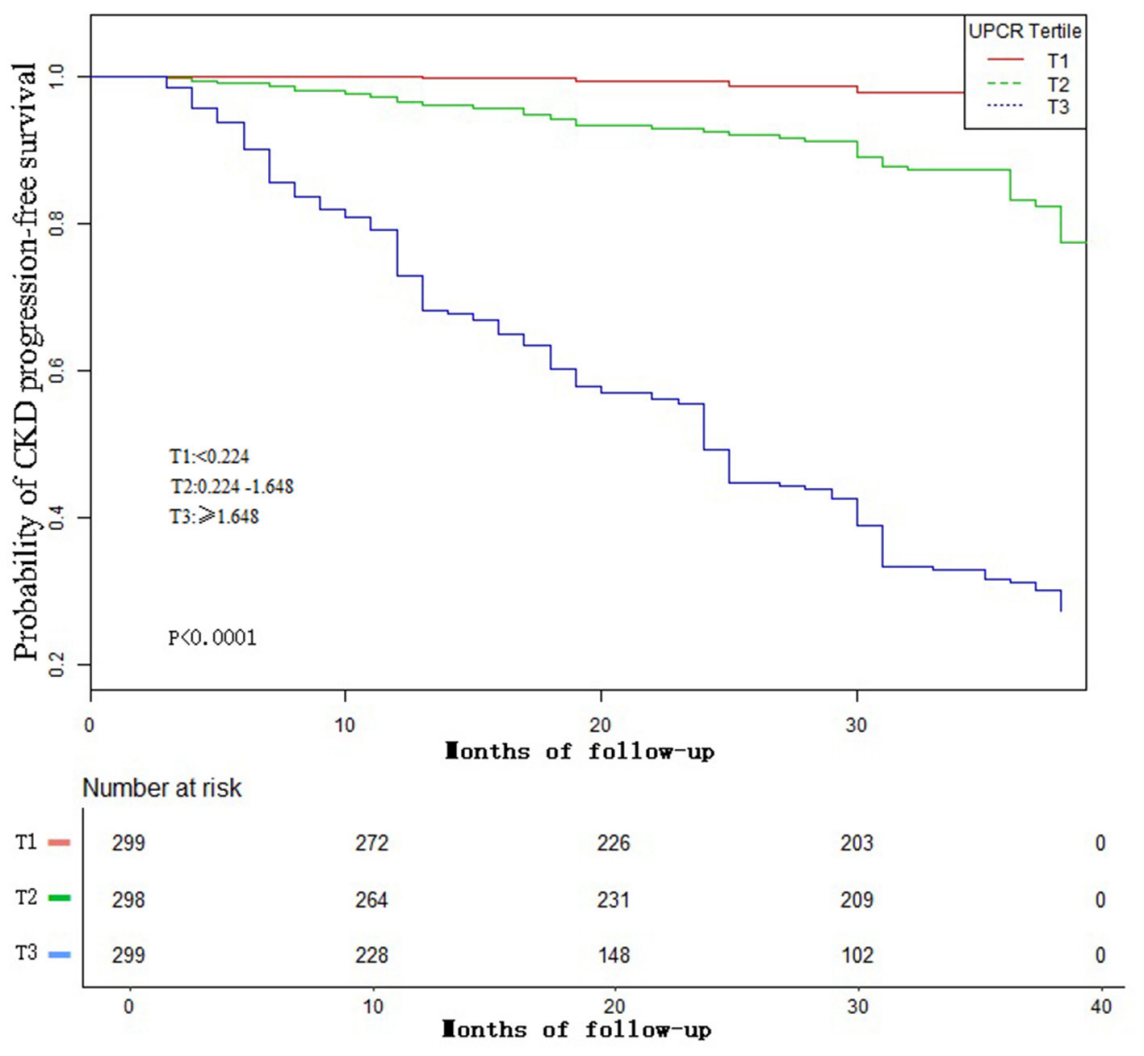
Kaplan–Meier event-free survival curve. Kaplan–Meier analysis of incident CKD progression-free survival based on UPCR groups (log-rank, *P* <0.0001).

Figure 4 showed the Kaplan-Meier curves of the probability of CKD progression-free survival stratified by UPCR categories. The probability of CKD progression-free survival between the three UPCR groups was significantly different (log-rank test, *p* < 0.0001). The probability of CKD progression-free survival gradually decreased with the increasing UPCR group, indicating that the higher the UPCR group, the higher the risk of CKD progression.

### The Results of the Relationship Between UPCR and CKD Progression

In this study, we constructed three models to analyze the independent effects of UPCR on CKD progression (univariate and multivariate Cox proportional hazard model). The effect sizes hazard ratio (HR) and 95% confidence intervals were listed in Table 4. In crude model, UPCR showed a positive association with CKD progression [HR = 1.210, 95% confidence interval (CI):1.760–1.903, *P* < 0.00001]. In the minimally adjusted model (adjusted gender, age, SBP, BMI, hypertension, diabetes, history of CVD, and etiology of CKD), the result did not have obvious change (HR: 1.180, 95% CI: 1.146–1.251). In the fully adjusted model (model II) (adjusted gender, age, SBP, BMI, hypertension, diabetes, history of CVD, etiology of CKD, HB, eGFR, ALB, urinary occult blood, use of calcium channel blocker, use of RAAS inhibitor, and use of diuretics), we could also detect the connection (HR = 1.164, 95% CI: 1.116–1.215, *P* < 0.00001). Namely, for each additional 1 unit of UPCR, the risk of CKD progression increased by 16.4%.

**Table 4:** Relationship between UPCR and the chronic kidney disease progression in different models.

**Variable**	**Crude model (HR, 95% CI, *P*)**	**Model I (HR, 95% CI, *P*)**	**Model II (HR, 95% CI, *P*)**
UPCR	1.210 (1.184, 1.236) <0.00001	1.180 (1.146, 1.215) <0.00001	1.164 (1.116, 1.215) <0.00001
**UPCR(Tertile)**
T1	Ref.	Ref.	Ref.
T2	6.007 (2.699, 13.372) 0.00001	5.544 (2.474, 12.423) 0.00003	2.673 (1.180, 6.057) 0.01846
T3	39.760 (18.678, 84.639) <0.00001	27.965 (12.772, 61.228) <0.00001	9.618 (4.272, 21.652) <0.00001
*P* for trend	<0.00001	<0.00001	<0.00001

*Crude model: we did not adjust other covariants*.

*Model I: we adjust age, gender, BMI, SBP, hypertension, diabetes, history of CVD, etiology of CKD*.

*odel II: we adjust age, gender, BMI, SBP, hypertension, diabetes, history of CVD, etiology of CKD, HB, eGFR, ALB, urinary occult blood, use of RAAS inhibitor, use of calcium channel blocker, use of diuretics*.

*CI, confidence; Ref, reference*.

### The Analyses of the Non-linear Relationship

**Figure 5:**
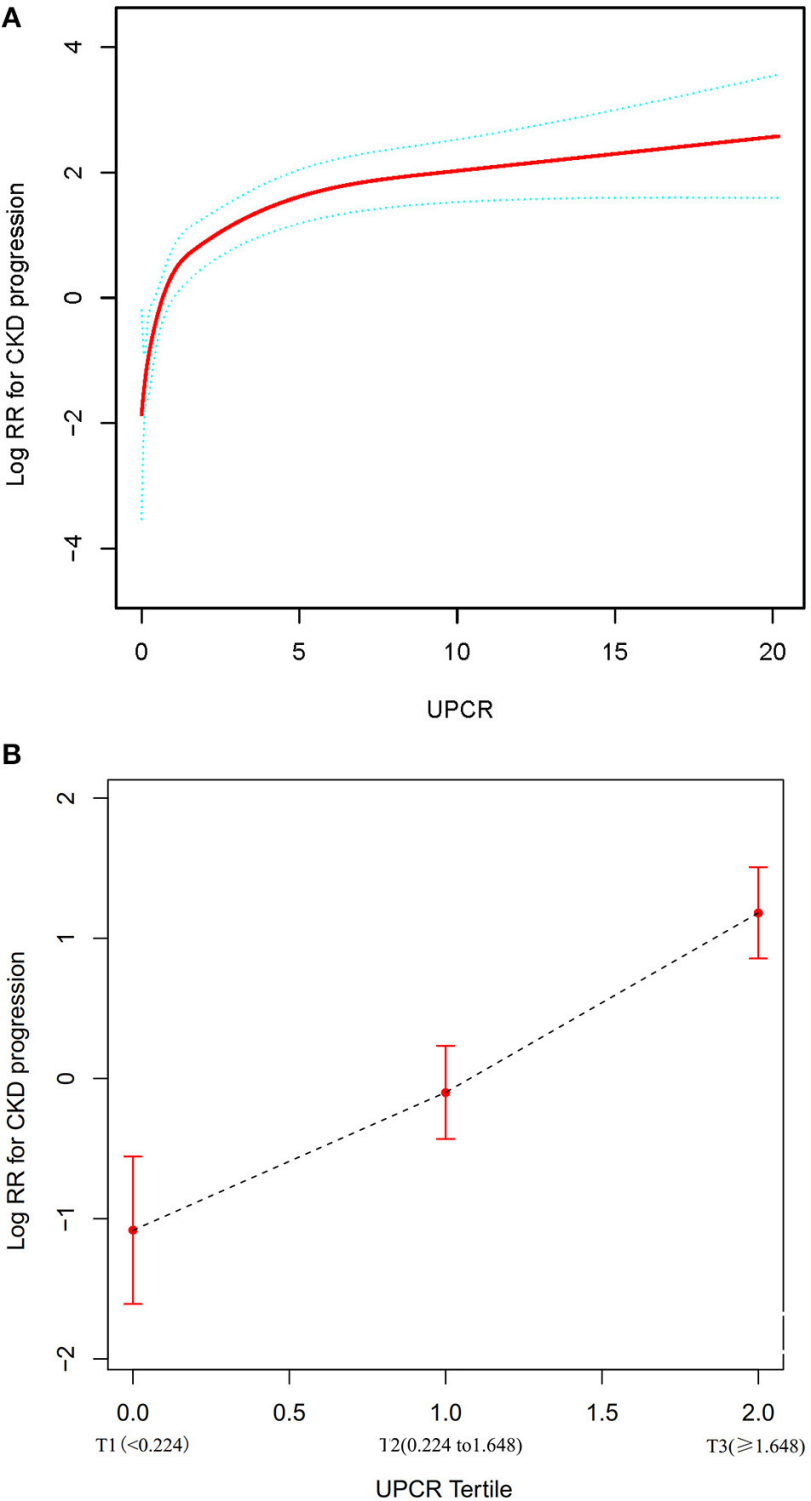
The non-linear relationship between UPCR and CKD progression. **(A)** The non-linear relationship between UPCR (continuous variable) and CKD progression. **(B)** The non-linear relationship between UPCR (tertile variable) and CKD progression. We used a Cox proportional hazards regression model with cubic spline functions and smooth curve fitting (penalized spline method) to evaluate the relationship between UPCR and incident CKD progression. The result showed that the relationship between UPCR and CKD progression was non-linear after adjusting for age, gender, BMI, SBP, hypertension, diabetes, history of CVD, etiology of CKD, HB, eGFR, ALB, urinary occult blood, use of RAAS inhibitor, use of calcium channel blocker and use of diuretics.

We used a Cox proportional hazards regression model with cubic spline functions to evaluate the relationship between UPCR (as continuous and tertile variables) and incident CKD progression (Figures 5A,B). The result showed that the relationship between UPCR and CKD progression was non-linear after adjusting for related confounding factors. When UPCR was a continuous variable, we used both the Cox proportional hazard model and the two-piecewise Cox proportional hazard model to fit the association and select the best fit model based on P for log-likelihood ratio test.

Because the *P* for the log-likelihood ratio test was <0.05, we chose the two-piecewise Cox proportional hazard model for fitting the association between UPCR and CKD progression because it could accurately represent the relationship. By the two-piecewise Cox proportional hazard model and recursive algorithm, we calculated the inflection point was 1.699. We observed a stronger positive association between UPCR and CKD progression on the left side of the inflection point, the HR and 95% CI were 4.377, 2.956–6.483, respectively. The results showed that a 1-unit increase in UPCR levels was associated with a 4.377-fold greater risk of CKD progression when the UCR was less than the 1.699. On the right side of the inflection point, we only observed a relatively weaker positive relationship between UPCR and CKD progression, the HR and 95% CI were 1.100, 1.049–1.153, respectively. Which indicated that a 1 unit increase in the UPCR level was only associated with a 1.1 times greater in the risk of CKD progression when UPCR > 1.699 (Table 5).

**Table 5:** The result of the two-piecewise linear regression model.

	**CKD progression**
	**(HR, 95% CI, *P*)**
Fitting model by standard linear regression	1.164 (1.116, 1.215) <0.0001
**Fitting model by two-piecewise linear regression**	
Inflection point of UPCR	1.699
≤ 1.699	4.377 (2.956, 6.483) <0.0001
>1.699	1.100 (1.049, 1.153) <0.0001
*P* for log-likelihood ratio test	<0.001

*CI, Confidence interval*.

*We adjusted age, gender, BMI, SBP, hypertension, diabetes, history of CVD, etiology of CKD, HB, eGFR, ALB, urinary occult blood, use of RAAS inhibitor, use of calcium channel blocker and use of diuretics*.

When UCPR was used as a tertile variable, we found that as its grade increased, there was a corresponding increase in the risk of CKD progression, with a more pronounced increase in T2–T3 than T1–T2 (Figure 5).

### Sensitivity Analysis

A series of sensitivity analyses were performed to confirm our findings' robustness. We first converted UPCR from a continuous variable to a categorical variable (according to tertile) and then put the categorical-transformed UPCR back into the model. The *P* for trend of UPCR as a categorical variable in the fully adjusted model was consistent with the result when UPCR was a continuous variable (*P* for trend <0.00001). Besides, we also found the trend of the effect size in different UPCR groups was non-equidistant.

In addition, we excluded patients with UPCR < 0.3 to explore the non-linear relationship between UPCR and CKD progression in other sensitivity analyses. The result showed that there was still a non-linear association between UPCR and CKD progression after adjusting for related confounding factors. By the two-piecewise Cox proportional hazard model and recursive algorithm, we calculated the inflection point was 1.607. We could still observe a stronger positive association between UPCR and CKD progression on the left side of the inflection point, and a relatively weaker positive relationship on the right side of the inflection point (Table 6, Supplementary Figure S1).

**Table 6:** The result of the two-piecewise linear regression model in patients with UCPR > 0.3 for sensitivity analyses.

	**CKD progression**
	**(HR, 95%CI, *P*)**
Fitting model by standard linear regression	1.148 (1.099, 1.199) <0.0001
**Fitting model by two-piecewise linear regression**	
Inflection point of UPCR	1.607
≤ 1.699	5.466 (3.031, 9.856) <0.0001
>1.699	1.101 (1.050, 1.155) <0.0001
*P* for log-likelihood ratio test	<0.001

*CI, Confidence interval*.

*We adjusted age, gender, BMI, SBP, hypertension, diabetes, history of CVD, etiology of CKD, HB, eGFR, ALB, urinary occult blood, use of RAAS inhibitor, use of calcium channel blocker and use of diuretics*.

### UPCR and Longitudinal eGFR

**Figure 6:**
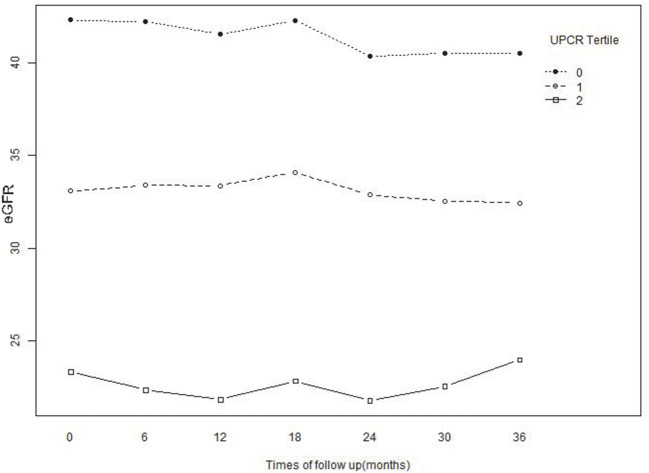
Baseline and predicted 3-year longitudinal changes in eGFR for the patients with UPCR at baseline were divided into three groups according to tertile. The patients whose baseline UPCR was in the lowest tertile showed an accelerated decrease in eGFR compared with the other two tertiles.

We took advantage of the repeated measurements of eGFR at each follow-up visit to characterize the longitudinal changes in eGFR over time in the cohort. Using a linear mixed-effects regression model, we evaluated baseline UPCR's profound influence on the longitudinal changes in eGFR. In this model, a statistically significant interaction term between time and UPCR indicated that the longitudinal changes in eGFR were influenced by UPCR. As shown in Table 7 and Figure 6, after adjusting for BMI, gender, age, SBP, hypertension, diabetes, history of CVD, etiology of CKD, HB, eGFR, ALB, urinary occult blood, use of calcium channel blocker, use of RAAS inhibitor, and use of diuretics, baseline UPCR was independently associated with the longitudinal changes in eGFR (*p* < 0.001 for the interaction term with time). Besides, the negative association between baseline UPCR and eGFR was also gradually increased with the increase of follow-up time. Each 1 unit of UPCR increased, eGFR decreased by 1.023 ml/min per 1.73 m^2^ at baseline, while half a year of follow-up, eGFR decreased by 0.489 ml/min per 1.73 m^2^ more than the level of decrease at baseline. After a year of follow-up, eGFR decreased by 0.936 ml/min per 1.73 m^2^ more than the baseline. The baseline UPCR had the greatest effect on the decline of eGFR after 30 months of follow-up. Specifically, eGFR decreased by 2.556 ml/min per 1.73 m^2^ after 30 months of follow-up with an increase of 1 unit of UPCR at baseline.

**Table 7:** Relationship between baseline UPCR and longitudinal eGFR derived from a linear mixed-effects regression model.

**Variable**	**Crude model (β, 95%CI, *P*)**	**Model I (β, 95% CI, *P*)**
UPCR(baseline)	−1.653 (−2.010, −1.296) <0.001	−1.023 (−1.405, −0.641) <0.001
UPCR ×6th month	−0.487 (−0.641, −0.333) <0.001	−0.489 (−0.643, −0.335) <0.001
UPCR ×12th month	−0.932 (−1.106, −0.757) <0.001	−0.936 (−1.110, −0.762) <0.001
UPCR ×18th month	−1.200 (−1.386, −1.014) <0.001	−1.208 (−1.393, −1.023) <0.001
UPCR ×24th month	−1.189 (−1.382, −0.996) <0.001	−1.199 (−1.391, −1.006) <0.001
UPCR ×30th month	−1.521 (−1.735, −1.307) <0.001	−1.533 (−1.747, −1.319) <0.001
UPCR ×36th month	−1.441 (−1.667, −1.214) <0.001	−1.452 (−1.678, −1.226) <0.001

*CI, Confidence interval*.

*We adjusted age, gender, BMI, SBP, hypertension, diabetes, history of CVD, etiology of CKD, HB, eGFR, ALB, urinary occult blood, use of RAAS inhibitor, use of calcium channel blocker and use of diuretics*.

Figure 6 illustrated the effect of the baseline UPCR group on the longitudinal changes of eGFR. While stratified based on the tertiles of UPCR, the association between UPCR and eGFR changes was correspondingly divided into three parts. As UPCR was negatively correlated with eGFR, the patients with the lowest tertile of UPCR at baseline had the highest levels of eGFR at baseline and during follow-up. However, eGFR decreased rapidly in patients with the lowest UPCR group at baseline compared with the other two groups.

## Discussion

Our findings indicated UPCR was positively associated with CKD progression after adjusting other covariates. Besides, we calculated the inflection point of UPCR was 1.699, and we found the trend of the effect sizes on the left and right sides of the inflection point was not consistent [left (HR = 4.377, 95% CI: 2.956–6.483, *P* < 0.0001); right (HR = 1.100, 95% CI: 1.049–1.153, *P* < 0.0001)]. The result suggested a turning point effect on the independent association between UPCR and CKD progression. Using the linear mixed-effects regression model, we found that the impact of baseline UCPR on changes in eGFR was different at different follow-up times.

Proteinuria has been proposed as a surrogate endpoint in clinical trials on CKD progression (27). Evaluation of proteinuria is inexpensive and straightforward. Some previous studies have probed the association between proteinuria and renal progression in CKD patients. Mass sample of 106,177 participants from the general Japanese population identified proteinuria as the most powerful predictor of ESKD risk over 10 years (28). A similar study in France showed the most potent independent risk factors of poor renal outcome were proteinuria ≥1 g/day [proportional hazard risk (HR) =23.7, *P* = 0.0001] (29). Another study suggested that baseline UPCR and systolic BP levels were independently associated with CKD progression in children with non-glomerular CKD (12). In a retrospective study of 438 adults with IgA nephropathy, Hong Zhang et al. found that urine ACR, UPCR, and 24-h UPE had a comparable association with severe clinical and histologic findings. All of them showed good performance in predicting IgAN progression (11). On the contrary, another study that assessed the relationship between UPCR, ACR, and 24-h UPE in 6,842 patients with CKD focusing on performance at thresholds of 0.5 and 1 g/day of proteinuria, found that UPCR was a more sensitive screening test than ACR to predict clinically relevant proteinuria. The study also found that the relationship between ACR and UPCR was non-linear. UPCR was highly correlated with 24-h urine protein (Spearman's rho = 0.91), though ACR also performed well (rho = 0.84) (13). However, most of these studies explored the relationship between proteinuria and CKD progression only using the logistic regression model or Cox proportional hazards regression model. They neither further analyzed the possible non-linear relationship between UPCR and CKD progression nor explored the effect of UCPR on eGFR changes. In our research, having a similar sample size, the Cox proportional hazard regression model showed a positive association between UPCR and CKD progression, consistent with those studies. Besides, we found a non-linear relationship between UPCR and CKD progression through multivariate Cox proportional hazards regression analysis with cubic spline functions model and smooth curve fitting (penalized spline method). We also found the inflection point was 1.699. When UPCR was <1.699, there was a stronger positive relationship between UPCR and CKD progression. A 1 unit increase in the UPCR level was associated with 4.377 times greater in the risk of CKD progression (HR = 4.377, 95% CI: 2.956–6.483). However, when UPCR > 1.699, a 1 unit increase in the UPCR level was only associated with a 1.1 times greater in the risk of CKD progression (HR = 1.100, 95% CI: 1.049–1.153). The reason might be that other variables in the participant's baseline also influenced the risk of CKD progression. It could be seen from Supplementary Table S1 that compared with the UPCR < 1.699 group, people with UPCR ≥ 1.699 generally had higher SBP, BMI levels, higher rates of urinary occult blood, hypertension, diabetes, history of CVD, RAAS inhibitor use, and use of diuretics. Besides, patients with UPCR ≥ 1.699 had a higher proportion of diabetic nephropathy as the primary disease. In contrast, patients generally had lower HB, ALB, and eGFR levels in the UPCR ≥ 1.699 group. However, the abnormality of the above indicators was closely related to the progress of CKD. When UPCR was >1.699, due to the presence of these CKD progression risk factors, UPCR had a relatively weak effect on the development of CKD progression. On the contrary, when UPCR was <1.699, the risk factors for CKD progressions, such as SBP, BMI, the proportion of hypertension, diabetes, and use of diuretics, were low. The impact on the occurrence of CKD progression was weakened; at this time, the effect of UPCR was relatively enhanced. It provided a further reference for the prevention of CKD progression in patients with different proteinuria states. The findings of this study should be helpful for future research on the establishment of diagnostic or predictive models of CKD progression. Combining the results in Tables 4, 5, it should be pointed out that since the distribution of UPCR was skewed, the impact of a 50% reduction in proteinuria in the high range when UPCR > 1.699 is bigger than a 50% reduction in the lower range when UPCR < 1.699. In addition, using a linear mixed-effects regression model, we assessed the profound effect of baseline UPCR on longitudinal changes in eGFR. We found that the impact of baseline UCPR on changes in eGFR was different at different follow-up times. With a 1 unit increase in the UPCR level, its effect on the decline in eGFR increased continuously with follow-up time. This result may help clinicians understand the impact of baseline proteinuria on short- and long-term renal outcomes in patients with CKD.

One of the currently widely accepted proteinuria-mediated progressive renal injury mechanisms involves a tubulointerstitial injury caused by the direct toxicity of filtered urine protein. Recent studies support that excessive protein accumulation in podocytes is a factor in the progressive damage of glomerular cells through the release of transforming growth factor-beta, which ultimately leads to myofibroblastic differentiation of mesangial cells (30). The underlying pathophysiological mechanism that has been proposed to link albuminuria to cardiovascular disease is peripheral vascular dysfunction, particularly renal endothelial dysfunction, accelerating the atherothrombotic process and thereby increasing cardiovascular risk (31). Early studies have concluded that clustered plasma proteins in the glomerular mesangial region can cause mesangial cell damage and proliferation, increasing mesangial matrix production and eventually aggravating glomerulosclerosis (32).

Our study has some strengths. (1) Compared with the previous research, the research on the non-linearity addressing was a significant improvement. (2) This observational study was susceptible to residual bias due to unmeasured confounding factors. Therefore, strict statistical adjustment was used to minimize residual confounders. (3) In this study, we tested the robustness of the results through a series of sensitivity analyses (target independent variable transformation, log-likelihood ratio test, and reanalyzing the association between UPCR and CKD progression after excluding patients with UPCR < 0.3, etc.) to ensure the reliability of the results. (4) Using a linear mixed-effects regression model, we assessed the profound effect of baseline UPCR on longitudinal changes in eGFR.

Our research has the following shortcomings and needs attention. First, the data was obtained from the study of the CKD-ROUTE in Japan, and the data has been screened by Iimori et al. (14). Therefore, we could not conclude whether our findings are suitable for people in other areas of a different race. Because this was secondary data analysis, factors not measured in the original study could not be adjusted, such as blood phosphorus level. Second, the attending doctor's diagnosis determined the etiology of CKD. Many patients did not undergo renal biopsy. Third, the effect of diet therapy was not evaluated by a specialized nephrologist. Fourth, the participants enrolled in the present study were patients with CKD stages 2–5. The eGFR of all patients was <90. For patients with CKD stage 1, the relationship between UPCR and CKD progression needs to be further explored. Fifth, the present study only measured UPCR, and other parameters at baseline did not consider changes of UPCR over time. In the future, we can consider designing our studies or collaborating with other researchers to collect as many variables as possible, including patients with CKD stages 1–5 and information on the evolution of UPCR during patients follow-up, to facilitate our better analysis of the impact of change in proteinuria on outcomes as well.

## Conclusion

This study demonstrates a positive and non-linear relationship between UPCR and incident CKD progression in the Japanese population. There is a threshold effect between the UPCR level and CKD progression. This result is expected to provide a reference for the clinicians to control UPCR. Reducing the UPCR level can significantly reduce the risk of CKD progression and slow down the decline in eGFR levels.

## Data Availability Statement

The datasets presented in this study can be found in online repositories. The names of the repository/repositories and accession number(s) can be found in the article/Supplementary Material.

## Ethics Statement

The studies involving human participants were reviewed and approved by the Ethical Committees of Tokyo Medical and Dental University, School of Medicine (No. 883). The patients/participants provided their written informed consent to participate in this study.

## Author Contributions

XQ, HH, JC, and XW contributed to the study concept and design, researched and interpreted the data, and drafted the manuscript. QW and ZW are the guarantors of this work and, as such, had full access to all the data in the study and took responsibility for the data's integrity and the accuracy of the data analysis. All authors read and approved the final manuscript.

## Funding

This study was supported in part by the Discipline Construction Ability Enhancement Project of the Shenzhen Municipal Health Commission (SZXJ2017031) and Shenzhen Key Medical Discipline Construction Fund (SZXK009).

## Conflict of Interest

The authors declare that the research was conducted in the absence of any commercial or financial relationships that could be construed as a potential conflict of interest.

## Publisher's Note

All claims expressed in this article are solely those of the authors and do not necessarily represent those of their affiliated organizations, or those of the publisher, the editors and the reviewers. Any product that may be evaluated in this article, or claim that may be made by its manufacturer, is not guaranteed or endorsed by the publisher.

## Abbreviations

UPCR: urinary protein-to-creatinine ratio
CKD: chronic kidney disease
CKD-ROUTE: chronic kidney disease research of outcomes in treatment and epidemiology
KDIGO: Kidney Disease Improving Global Outcomes
ESRD: end-stage renal disease
ACR: albumin-creatinine ratio
UPE: urine protein excretion
BMI: body mass index
SBP: systolic blood pressure
DBP: diastolic blood pressure
Scr: serum creatinine
eGFR: estimated glomerular filtration rate
ALB: serum albumin
Hb: hemoglobin
CVD: cardiovascular disease
ARB: angiotensin receptor blockers
ACEI: angiotensin-converting enzyme inhibitors
AP: angina pectoris
MI: myocardial infarction
CHF: congestive heart failure
PAD: peripheral arterial disease.
