# Supplementary figures and images for: Association Between Urinary Protein-to-Creatinine Ratio and Chronic Kidney Disease Progression: A Secondary Analysis of a Prospective Cohort Study
Source: Front Med (Lausanne). 2022 Mar 31;9:854300. doi: 10.3389/fmed.2022.854300 (PMC9008575; doi:10.3389/fmed.2022.854300)

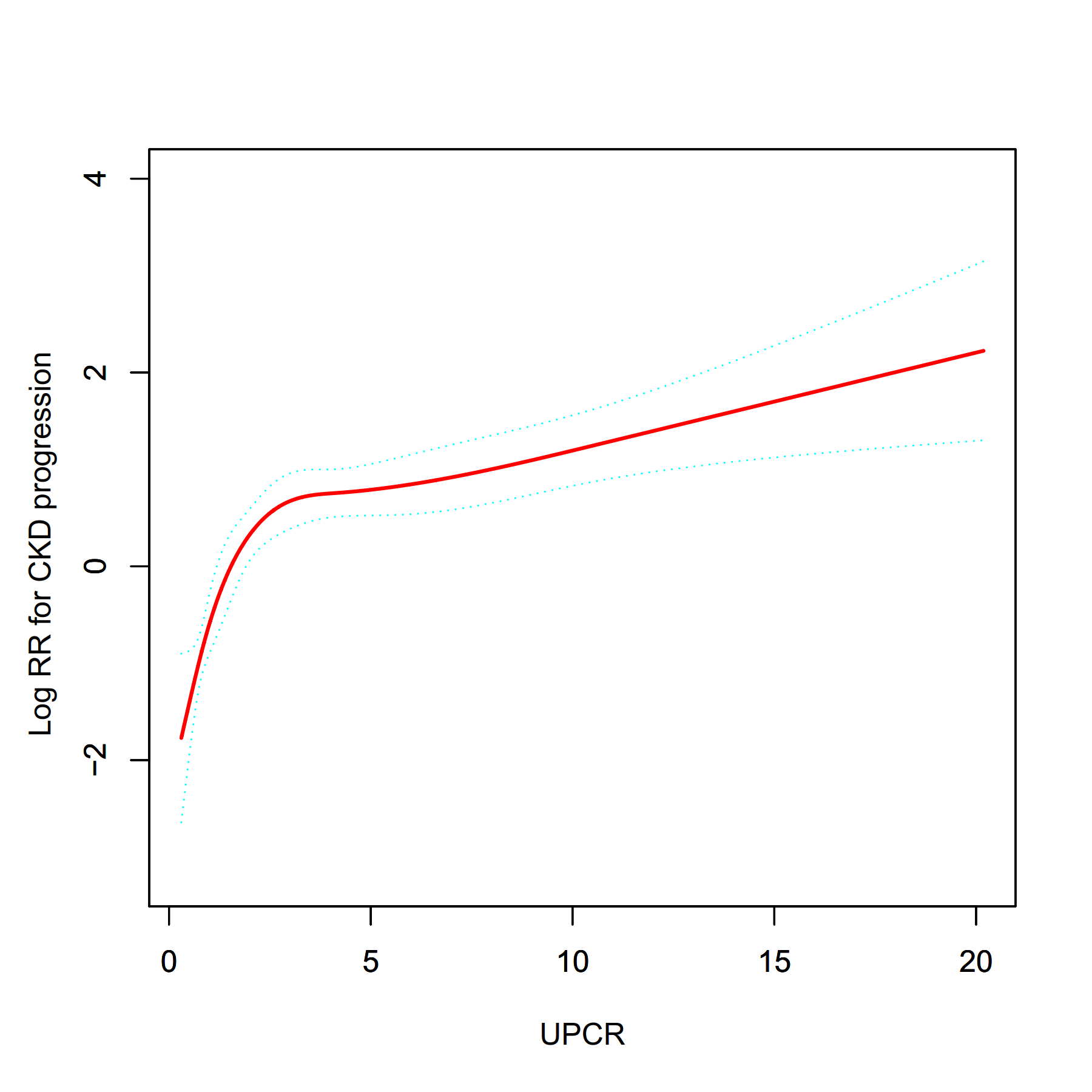

Supplement: Supplementary file 1 [file Image_1.TIF]
