# Supplementary material for: Association Between Urinary Protein-to-Creatinine Ratio and Chronic Kidney Disease Progression: A Secondary Analysis of a Prospective Cohort Study
Source: Front Med (Lausanne). 2022 Mar 31;9:854300. doi: 10.3389/fmed.2022.854300 (PMC9008575; doi:10.3389/fmed.2022.854300)
Supplement: Supplementary file 2 [file Table_1.DOCX]

**Association between urinary protein to creatinine ratio and chronic kidney disease progression: a secondary analysis of a prospective cohort study**

**Running title: UPCR and CKD progression**

**Xun QIN^#1^, Haofei HU^#2,3,4^, Ji CEN^#1^, Xiaoyu WANG^#1^, Qijun WAN^2,3,4*^, Zhe WEI^1*^**

^1^Department of Nephrology, Hechi People's Hospital, Hechi 547000, Guangxi Zhuang Autonomous Region, China

^2^Department of Nephrology, Shenzhen Second People’s Hospital, Shenzhen 518000, Guangdong Province, China

^3^Department of Nephrology, The First Affiliated Hospital of Shenzhen University, Shenzhen 518000, Guangdong Province, China

^4^Shenzhen University Health Science Center, Shenzhen 518000, Guangdong Province, China

**^#^**Xun QIN, Haofei HU, Ji CEN and Xiaoyu WANG have contributed equally to this work.

*Corresponding author

**Qijun WAN**

Department of Nephrology,

Shenzhen Second People’s Hospital,

No.3002 Sungang Road, Futian District,

Shenzhen 518000,

Guangdong Province,

China

Tel:+86-755-83366388

E-mail: [wanqijun12345@126.com](mailto:wanqijun12345@126.com)

*Corresponding author

**ZheWEI**

Department of Nephrology,

Hechi People's Hospital,

No. 455 Jincheng Middle Road, Jinchengjiang District,

Hechi 547000,

Guangxi Zhuang Autonomous Region,

China

Tel:+86-778-2293900

E-mail: 412429064@qq.com

**TableS1 The Baseline Characteristics of participants on both sides of the inflection point.**

| UPCR | <1.699 | >=1.699 | P-value |
| --- | --- | --- | --- |
| Participants | 603 | 293 |  |
| Age(years) | 68.1 ± 13.4 | 65.3 ± 13.3 | 0.004 |
| HB(g/dL) | 12.5 ± 2.2 | 11.3 ± 2.1 | <0.001 |
| eGFR (ml/min per 1.73 m2) | 37.3 ± 17.4 | 24.7 ± 16.1 | <0.001 |
| SBP(mmHg) | 135.5 ± 20.4 | 148.8 ± 22.7 | <0.001 |
| BMI(kg/m^2^) | 23.6 ± 3.6 | 24.3 ± 4.2 | 0.009 |
| ALB(g/dL) | 4.1 ± 0.5 | 3.4 ± 0.6 | <0.001 |
| Gender |  |  | 0.376 |
| Male | 429 (71.1%) | 200 (68.3%) |  |
| Female | 174 (28.9%) | 93 (31.7%) |  |
| Use of diuretics, n (%) | 157 (26.0%) | 134 (45.7%) | <0.001 |
| Use of calcium channel blocker, n (%) | 253 (42.0%) | 178 (60.8%) | <0.001 |
| Use of RAAS inhibitor, n(%) | 365 (60.5%) | 221 (75.4%) | <0.001 |
| Etiology of CKD |  |  | <0.001 |
| Diabetic nephropathy, n(%) | 74 (12.3%) | 158 (53.9%) |  |
| Nephrosclerosis, n (%) | 299 (49.6%) | 58 (19.8%) |  |
| Glomerulonephritis, n (%) | 106 (17.6%) | 57 (19.5%) |  |
| Other, n (%) | 124 (20.6%) | 20 (6.8%) |  |
| Hypertension, n (%) | 520 (86.2%) | 286 (97.6%) | <0.001 |
| Diabetes, n (%) | 169 (28.0%) | 176 (60.1%) | <0.001 |
| History of CVD, n (%) | 142 (23.5%) | 99 (33.8%) | 0.001 |
| Urinary occult blood, n(%) | 151 (25.1%) | 139 (47.4%) | <0.001 |

Continuous variables are presented as mean ± standard deviation and median with interquartile ranges. Categorical data are presented as numbers and percentages.

Abbreviations: BMI, body mass index; SBP, Systolic blood pressure; ALB, Serum albumin; HB, Hemoglobin; CKD, chronic kidney disease; CVD, cardiovascular disease; eGFR, estimated glomerular filtration rate; UPCR, urinary protein/creatinine ratio; g/gCr, gram per gram creatinine; RAAS, renin angiotensin aldosterone system.

**Figure S1. The non-linear relationship between UPCR and CKD progression in patients with UPCR>0.3**

We used a Cox proportional hazards regression model with cubic spline functions and smooth curve fitting (penalized spline method) to evaluate the relationship between UPCR and incident CKD progression in patients with UCPR>0.3. The result showed that the relationship between UPCR and CKD progression was still non-linear after adjusting for age, gender, BMI, SBP, hypertension, diabetes, history of CVD, etiology of CKD, HB, eGFR, ALB, urinary occult blood, use of RAAS inhibitor, use of calcium channel blocker and use of diuretics.
